# 

**Published:** 1890-06

**Authors:** 


JUNE, 1890.

Vol. VIII. No. 28.

THE

### BRISTOL MEDICO-CHIRURGICAL JOURNAL

A Quarterly Journal of the Medical Sciences for the West of England and South Wales

*PUBLISHED UNDER THE AUSPICES OF*

*THE BRISTOL MEDICO-CHIRURGICAL SOCIETY.*

EDITOR:

J. GREIG SMITH, M.A., F.R.S.E.

ASSISTANT-EDITOR:

L. M. GRIFFITHS.

LIBRARY OF CONGRESS
374777
DEC 1 1897
SMITHSONIAN DEPOSIT.

"Scire est nescire, nisi id me

Scire alius sciret."

BRISTOL: J. W. ARROWSMITH;
LONDON: J. & A. CHURCHILL, 11 NEW BURLINGTON STREET.

*Price One Shilling and Sixpence.*

